# PaintorPipe: a pipeline for genetic variant fine-mapping using functional annotations

**DOI:** 10.1093/bioadv/vbad188

**Published:** 2023-12-21

**Authors:** Zoé Gerber, Michel Fisun, Hugues Aschard, Sarah Djebali

**Affiliations:** IRSD, Université de Toulouse, INSERM, INRAE, ENVT, Université Toulouse III - Paul Sabatier (UPS), 31024 Toulouse, France; Bordeaux Bioinformatics Master, Université de Bordeaux, 33405 Talence, France; Department of Computational Biology, Pasteur Institute, Université Paris Cité, 75015 Paris, France; Department of Computational Biology, Pasteur Institute, Université Paris Cité, 75015 Paris, France; Program in Genetic Epidemiology and Statistical Genetics, Harvard T.H. Chan School of Public Health, Boston, MA 02115, United States; IRSD, Université de Toulouse, INSERM, INRAE, ENVT, Université Toulouse III - Paul Sabatier (UPS), 31024 Toulouse, France

## Abstract

**Motivation:**

Genome-wide association studies (GWAS) have identified thousands of genetic variants associated with common diseases. These results include a mix of causal and non-causal variants related through strong linkage disequilibrium (LD, i.e. highly correlated). Fine-mapping methods have been developed to decipher the causal from non-causal variants using GWAS results and LD information, assigning to each variant a probability of being causal. In this field, the PAINTOR program has become a standard, one of its advantages being its ability to take into account functional annotations. This approach requires many pre- and post-processing steps. Here, we developed a Nextflow pipeline called PaintorPipe that wraps all these steps and the fine-mapping itself together. PaintorPipe uses three independent sources of information: GWAS summary statistics, LD information and functional annotations, to rank the variants according to their susceptibility to be involved in the disease development. The PAINTOR framework is used to calculate the posterior probability of each variant (single nucleotide polymorphism) to be causal (a.k.a. Bayesian fine-mapping). The resulting credible sets of variants are annotated with their biological functions and visualized using CANVIS. This pipeline requires minimal input from users (a GWAS summary statistics file and a set of functional annotation files) and is designed to be modular and customizable, allowing for an easy integration of diverse functional annotations.

**Availability and implementation:**

PaintorPipe is implemented in the Nextflow pipeline specific language, can be run locally or on a slurm cluster and handles containerization using Singularity. PaintorPipe is freely available on GitHub (https://github.com/sdjebali/PaintorPipe).

## 1 Introduction

Most common human diseases, such as diabetes, obesity, cardiovascular disease/stroke, cancer, neuropsychiatric, auto-immunity, and inflammatory diseases, are multifactorial, depending on numerous environmental and genetic factors. The genetic component is typically highly polygenic, likely involving hundreds to thousands of causal variants spread across the entire genome. The identification of genetic variants, mostly single nucleotide polymorphisms (SNPs), associated with those common polygenic diseases heavily relies on genome-wide association studies (GWAS). The GWAS consists in a massively parallel univariate testing of millions of variants using a standard linear additive model. This approach has proven quite efficient, identifying thousands of associations, but also has some limitations. In particular, the extent of linkage disequilibrium (LD, i.e. correlation) across variants in the human genome implies that numerous non-causal variants can capture signal through their correlation with causal ones. Deciphering the causal variants from non-causal ones found associated because of LD, has proven extremely difficult.

To address this challenge, *in silico* fine-mapping methods have been developed (Schaid *et al.* 2018) to prioritize genetic variants more likely to be causal. Most fine-mapping methods are built on a Bayesian framework which jointly model the effects of multiple SNPs on a trait and use LD information to assign a probability of causality to each variant (posterior probability, PP). PAINTOR (Kichaev *et al.* 2014) is one of such Bayesian fine-mapping methods. It is a popular approach that has been used for the prioritization of variants in hundreds of fine-mapping studies. One advantage of PAINTOR is its ability to incorporate functional annotations. It builds on the assumption that causal variants might act through similar biological pathways, so that enrichment for a given annotation might be leveraged to improve the accuracy of fine-mapping results. However, running PAINTOR requires several pre- and post-processing steps, making it time-consuming and cumbersome.

In this paper, we describe the Nextflow (Di Tommaso *et al.* 2017) pipeline called PaintorPipe, that aims at overcoming this issue for fast and efficient fine-mapping analyses. PaintorPipe performs all the pre- and post-processing steps required for a fine-mapping, apply PAINTOR, and produce a summary results using existing tools directly from the PAINTOR output.

## 2 Workflow

**Figure 1.**
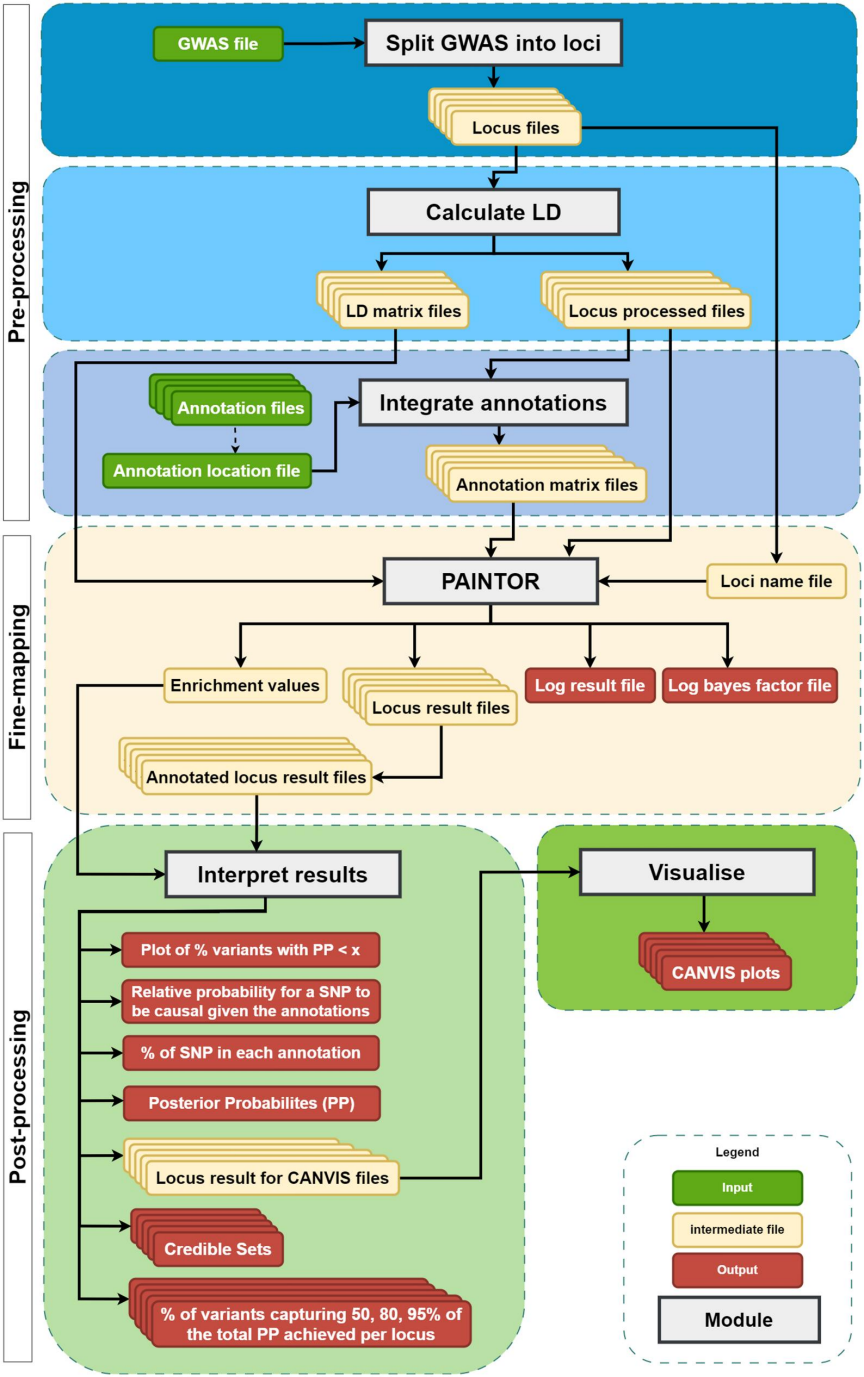
The PaintorPipe Workflow. The PaintorPipe workflow consists of three main parts, indicated on the left of the figure: pre-processing, fine-mapping, and post-processing. Each part is itself divided into modules represented as large horizontal boxes. The pre-processing part is made of three modules: *Split GWAS into loci*, *Calculate LD*, and *Integrate annotation*; the fine-mapping part is made of a single PAINTOR module; and the post-processing part is made of two modules, *Interpret results*, and *Visualise* that uses the CANVIS tool. In practice, each module is composed of several Nextflow processes, but for clarity purposes, only the module name is indicated on the figure. The required input files, including the GWAS file and the functional annotation files, are represented as small green boxes, while the intermediate files, that are used by other steps of the pipeline and that can optionally be saved, are represented as small yellow boxes. Finally, the output files are represented as small red boxes.

The PaintorPipe workflow is divided into three main parts: Pre-processing, Fine-mapping, and Post-Processing (Fig. 1), that are described below.

### 2.1 Pre-processing

The pre-processing part aims at preparing input files for running PAINTOR and involves three modules: Split GWAS into loci, Calculate LD, and Integrate annotations.

#### 2.1.1 Split GWAS into loci

The GWAS dataset is first divided into loci. The SNPs are first split into chromosomes, and ordered by ascending *P*-value. The loci are then generated per chromosome, starting from the smallest *P*-value SNP and iteratively until no SNP with a *P*-value lower than pval_lead (5e−8 by default) is found. More precisely the smallest *P*-value SNP is selected and grouped with all other SNPs with *P*-values lower than pval_nonlead (1 by default) and located less than a distance away from it (500 kilobases by default). This process is repeated with the second smallest *P*-value SNP, and if the newly formed locus overlaps with the first one, they are merged together. This is done iteratively and when a new locus is formed, its position on the genome is compared to the ones of all previously made loci and in case of overlap they are merged together. Therefore the final loci are completely disjoint on the genome, i.e. do not share any SNPs. The resulting loci are provided as SNP files in which each SNP is associated to multiple pieces of information including a *z-score*, computed by dividing the SNP effect size by the SNP effect size standard error.

#### 2.1.2 Calculate LD

Each resulting locus file is subsequently passed on to the *Calculate LD* module that produces a linkage disequilibrium (LD) matrix for each locus, by computing the Pearson correlation coefficient between each pair of SNPs of the locus. In addition to the locus SNPs, this module takes as input a map file and variant VCF files that are automatically downloaded from the 1000 Genomes Project website (https://www.internationalgenome.org/, Finucane *et al.* 2015).

#### 2.1.3 Integrate annotations

Since the majority of disease associated variants are found in non-coding regions of the genome (Claringbould and Zaugg 2021), a functional annotation of these variants is crucial. Variants located in regulatory regions, such as enhancers or promoters, can influence gene expression and increase the risk of developing the disease (Claringbould and Zaugg 2021).

**Figure 2.**
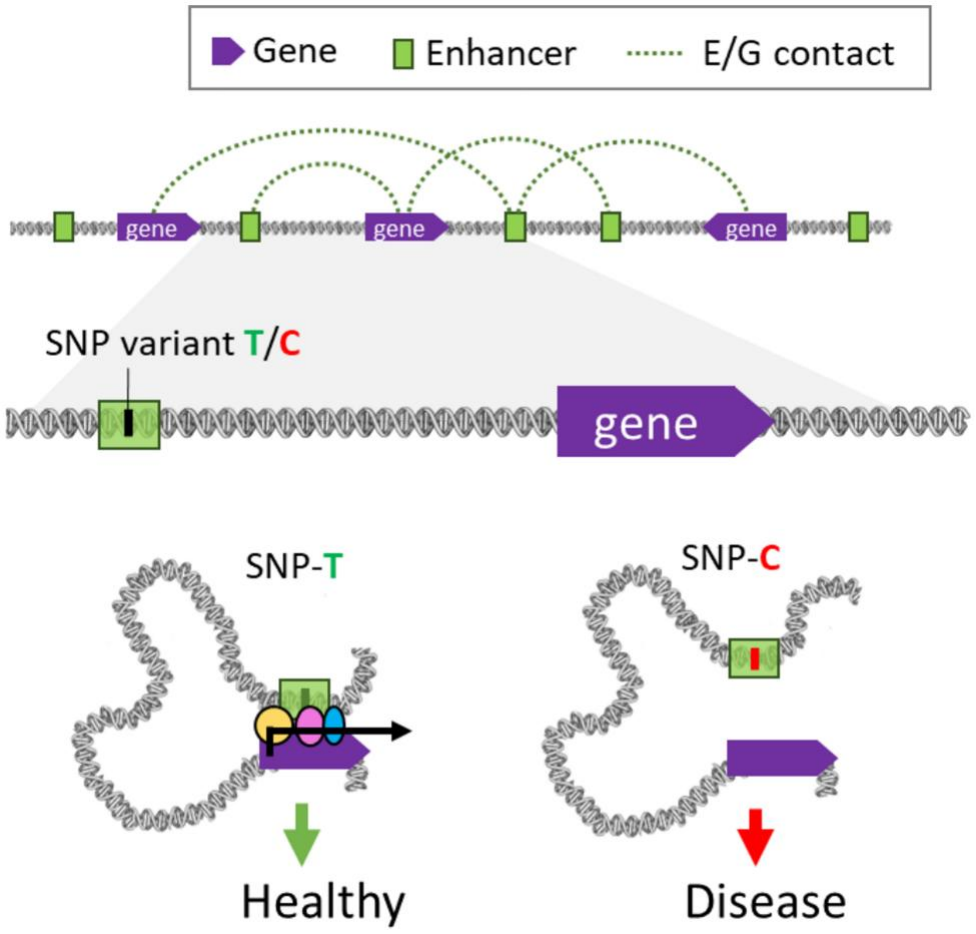
Illustration of the effect of genetic variants located in gene enhancers. (Top) Each enhancer can be linked to multiple genes, and conversely each gene can be linked to multiple enhancers. (Bottom) Here, an SNP variant located in an enhancer disrupts the activity of a distant gene, leading to increased disease risk.

Therefore, functional annotations, which provide information on the biological functions of different genomic regions, can help prioritize disease-causing variants by increasing their weight. In addition, functional annotations can also be used *a posteriori*, to better understand the mechanisms underlying diseases, which can lead to the development of new treatments or more effective preventive strategies. An example of possible disease-causing mechanism is shown in Fig. 2.

Within PaintorPipe, the *Integrate annotation* module only requires a single input file that provides the short names and the file paths on the system, of the different annotations. This process then computes the overlap between each SNP and each annotation, leading to an annotation matrix for each locus with SNPs in rows and annotations in columns. These matrices are made of 0 and 1 values, depending on whether the SNP is included in the given annotation. To achieve optimal results with the PAINTOR framework and as stated by its authors, it is recommended to use a maximum of five annotations.

### 2.2 Fine-mapping

The PP of causality of each variant is calculated by PAINTOR using the outputs of the pre-processing modules (i.e. the loci, the LD matrices and the annotation matrices). Note that the running time for this module can be computationally expensive (several hours), especially for datasets with a large number of variants (millions). For each locus, PAINTOR identifies credible sets of SNPs that have a high probability of being causal and are likely to contain the causal variants. Once the fine-mapping analysis is performed, PaintorPipe retrieves results from each locus and annotates them for a better interpretation. This is the Post-processing part described below.

### 2.3 Post-processing

**Figure 3.**
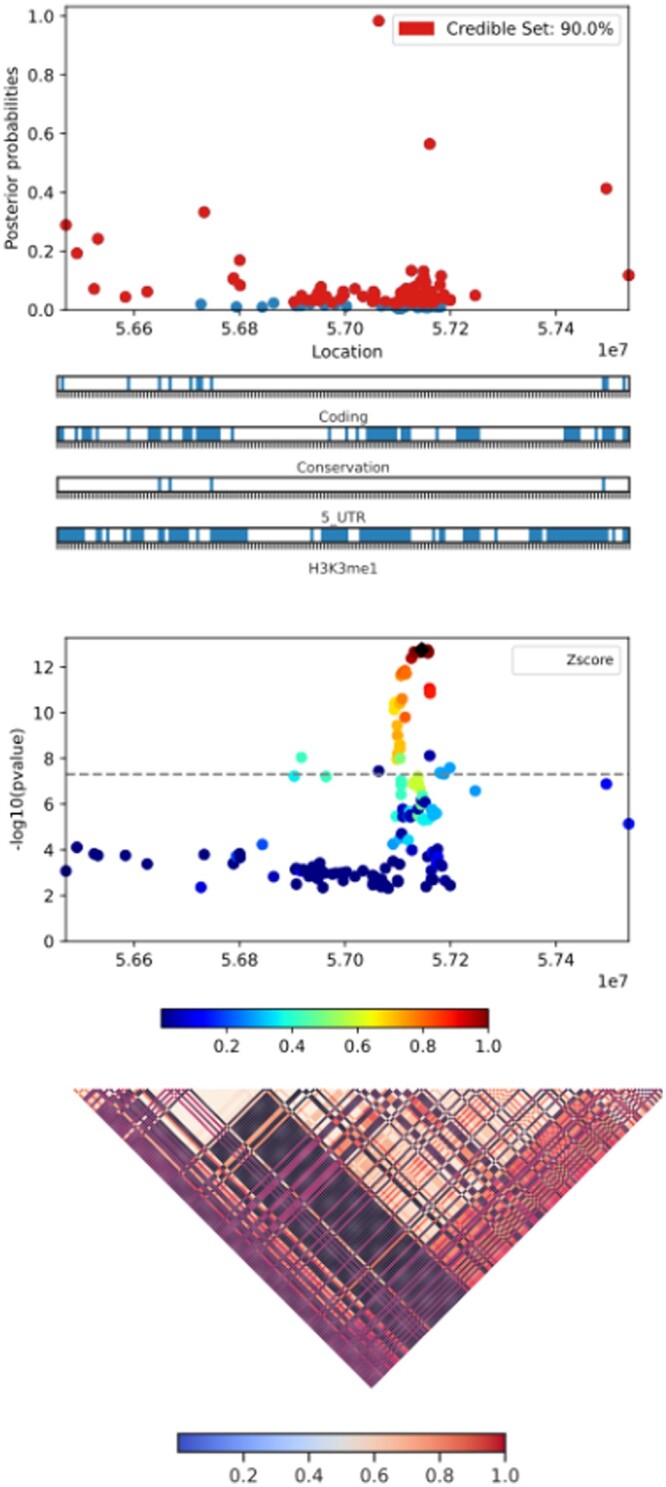
Example of a CANVIS visualization produced by PaintorPipe on a specific locus. The plot is made of three parts (from top to bottom): (i) a dotplot showing for each SNP of the locus, its genomic position on the *x* axis and its posterior probability to be causal (PP) on the *y* axis, with the credible set highlighted in red. The non-causal SNPs (not significant) are plotted in blue, and the different types of annotations used in this run are indicated by horizontal bars below the plot; (ii) a dotplot representing for each SNP of the locus, its genomic position on the x axis and its −log10(*P* value) on the *y* axis. The most probable causal variant is represented by a black diamond, and the color gradient represents the linkage disequilibrium (correlation) between this variant and the other SNPs of the locus; (iii) a heatmap corresponding to the linkage disequilibrium (SNP pair correlation) of the locus. Here, PaintorPipe version 1.2 was run with the following parameters −−chromosome_header Chr −− pvalue_nonlead 0.01−− snp 100000 on the 2018 Coronary Atery Disease (CAD) GWAS study Van Der Harst and Verweij (2018) using four annotations from Finucane *et al.* (2015). This run produced 149 loci. This figure presents the sixth locus of chromosome 6.

The post-processing part produces plots and tables for an easier interpretation of the fine-mapping results. Several computations are performed, including the number and percentage of all (or each locus) SNPs reaching 50%, 80%, and 95% of the cumulative sum of all (or each locus) SNP PPs. Finally, thanks to the CANVIS tool, each credible set of SNPs within a locus is graphically represented, together with the LD structure of the locus (Fig. 3).

## 3 Details and parameters

PaintorPipe is designed to work on human data and to be customizable with several adjustable parameters. Only two inputs are required to run the pipeline: the GWAS summary statistics file (using the—gwasFile parameter) and the annotation location file (using the—annotationsFile parameter).

The other input and output parameters are optional and have default values. This includes in particular the header parameters of the GWAS file, the different adjustable thresholds for the *P*-values and the posterior probabilities, but also the version of the reference genome (hg19 or hg38) and the distance in kilobases (kb) around the lead SNP used to define a locus (see Pre-processing section above). As the pipeline is implemented in Nextflow, it is possible to use Nextflow optional parameters (in particular the –C option to provide a custom configuration file).

In order to ensure reproducibility and manage tools, packages and versions in a controlled environment, PaintorPipe uses a Singularity image (Kurtzer *et al.* 2017). This Singularity image is hosted on Sylabs (https://sylabs.io/), and is specified in the configuration file. The pipeline can be run on a local machine or on a slurm cluster, and can therefore take advantage of the increased resources available on the latter to speed up the analyses (e.g. over 24 h for a GWAS file of 7 million SNPs).

## 4 Conclusion

PAINTOR is a popular fine-mapping software, that uses a Bayesian approach to calculate the posterior probability of SNPs to be causal. Its ability to incorporate functional annotations makes it a powerful tool for identifying causal variants underlying polygenic traits and diseases. To address its time-consuming and burdensome pre- and post-processing parts, we developed PaintorPipe, a Nextflow DSL2 pipeline that automates the entire process for fast and user-friendly analysis. By reducing the number of candidate variants to a more manageable and meaningful set, PaintorPipe can be used to prioritize candidate variants for further investigation.

Indeed, the variants with the highest probability of being causal can be more carefully inspected before experimental validation. For instance their location with respect to genes and regulatory elements can be computed (or derived from the pipeline outputs in case such functional annotations were used) and for the case of variants located in enhancers, the genes they are susceptible to affect can be retrieved from predicted enhancer/gene relationships [for instance from the ABC model (Fulco *et al.* 2019, Nasser *et al.* 2021) at https://www.engreitzlab.org/resources].

In short, PaintorPipe greatly facilitates the use of the PAINTOR fine-mapping program, allowing to run it from a minimum set of inputs (GWAS and annotation location files) in a reproducible way, and producing exploitable results for interpretation and visualization.

Finally, while we focused our development on PAINTOR, most fine-mapping methods use similar information (Z-scores, LD matrix across loci, and for some of them functional annotations), and produce similar outputs. We thus expect future versions of PaintorPipe to be able to use some of these other tools (Wang *et al.* 2020, Yang *et al.* 2023).

## Data Availability

The PaintorPipe code and small test dataset are freely available from GitHub at https://github.com/sdjebali/PaintorPipe.
